# Supplementary material for: Domain gain or loss in a fungal chitinase enables specialization towards antagonism or immune suppression
Source: Nat Commun. 2026 Mar 30;17:3115. doi: 10.1038/s41467-026-71064-0 (PMC13039781; doi:10.1038/s41467-026-71064-0)
Supplement: Supplementary file 1 — Supplementary Information [file 41467_2026_71064_MOESM1_ESM.pdf]

Supplementary information

Domain gain or loss in a fungal chitinase enables specialization towards antagonism or immune suppression

Ruben Eichfeld[1,2], Asmamaw B. Endeshaw[1], Margareta J. Hellmann[3], Taim Nassr[1], Bruno M. Moerschbacher[3], Alga Zuccaro[1,2*]

[1]University of Cologne, Institute for Plant Sciences, Cologne, Germany

[2]Cluster of Excellence on Plant Sciences (CEPLAS), Cologne, Germany

[3]University of Münster, Institute of Biology and Biotechnology of Plants, Münster, Germany

*Correspondence: azuccaro@uni-koeln.de

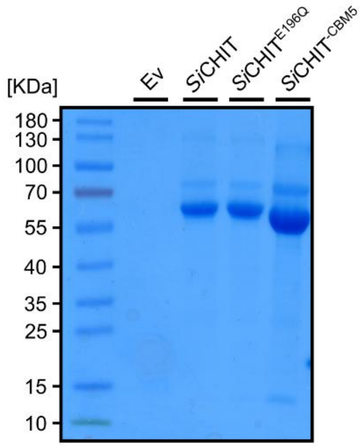

Supplementary Figure 1: Purification of chitinases. The coding sequences with an N-terminal His-Tag were cloned into an expression vector for *E. coli* (PQE80L) and induced with IPTG. The purification was conducted using Nickel-NTA slurry and purified proteins were visualized on a 10 % SDS-Gel and stained with Coomassie brilliant blue. Predicted sizes of purified proteins with His-tag: *Si*CHIT: 57.5 KDa; *Si*CHIT^{E196Q}: 57.5 KDa; *Si*CHIT^{-CBM5}: 52.2 KDa.

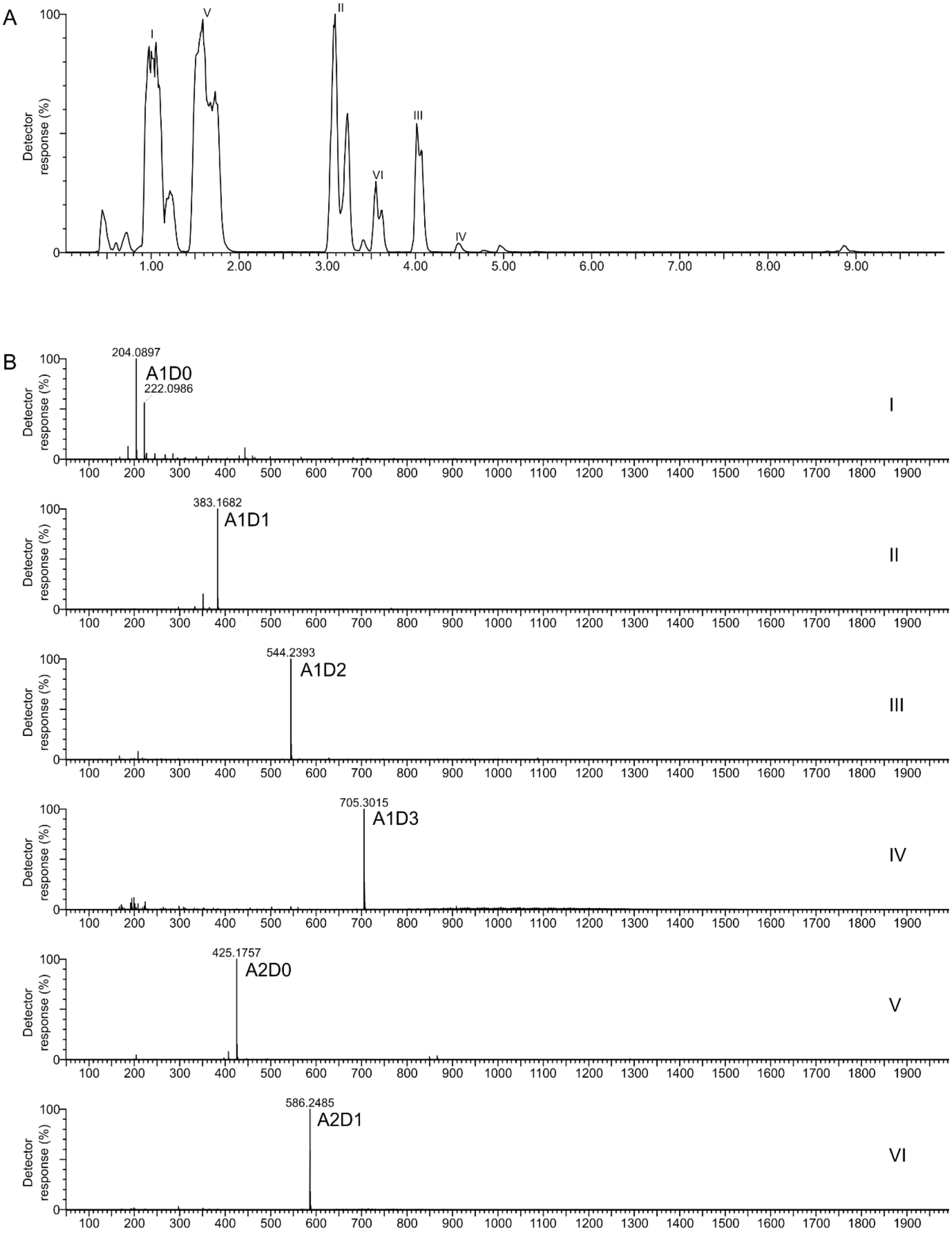

Supplementary Figure 2: Exemplary MS^1 data of the product profile of *Si*CHIT on crab shell chitin. A) Base peak chromatogram of the HILIC-ESI-MS^1 measurement of the small enzymatic products. **B)** Average mass spectra over each of the peaks labelled I-VI in **A**, the oligomers are identified based on the m/z value corresponding to the corresponding proton adduct of charge +1. A = acetylated, D = de-acetylated unit.

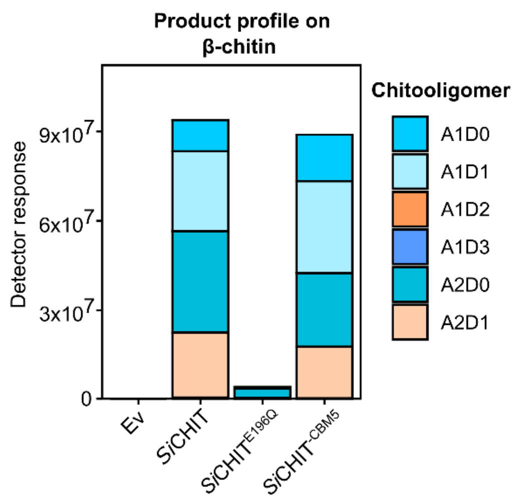

Supplementary Figure 3: Deletion of CBM5 does not affect product profile on β-chitin. Samples were incubated with 5 µM of chitinases for 24 h at 28 °C and hydrolysis products were identified and quantified via MS. A = acetylated, D = de-acetylated unit.

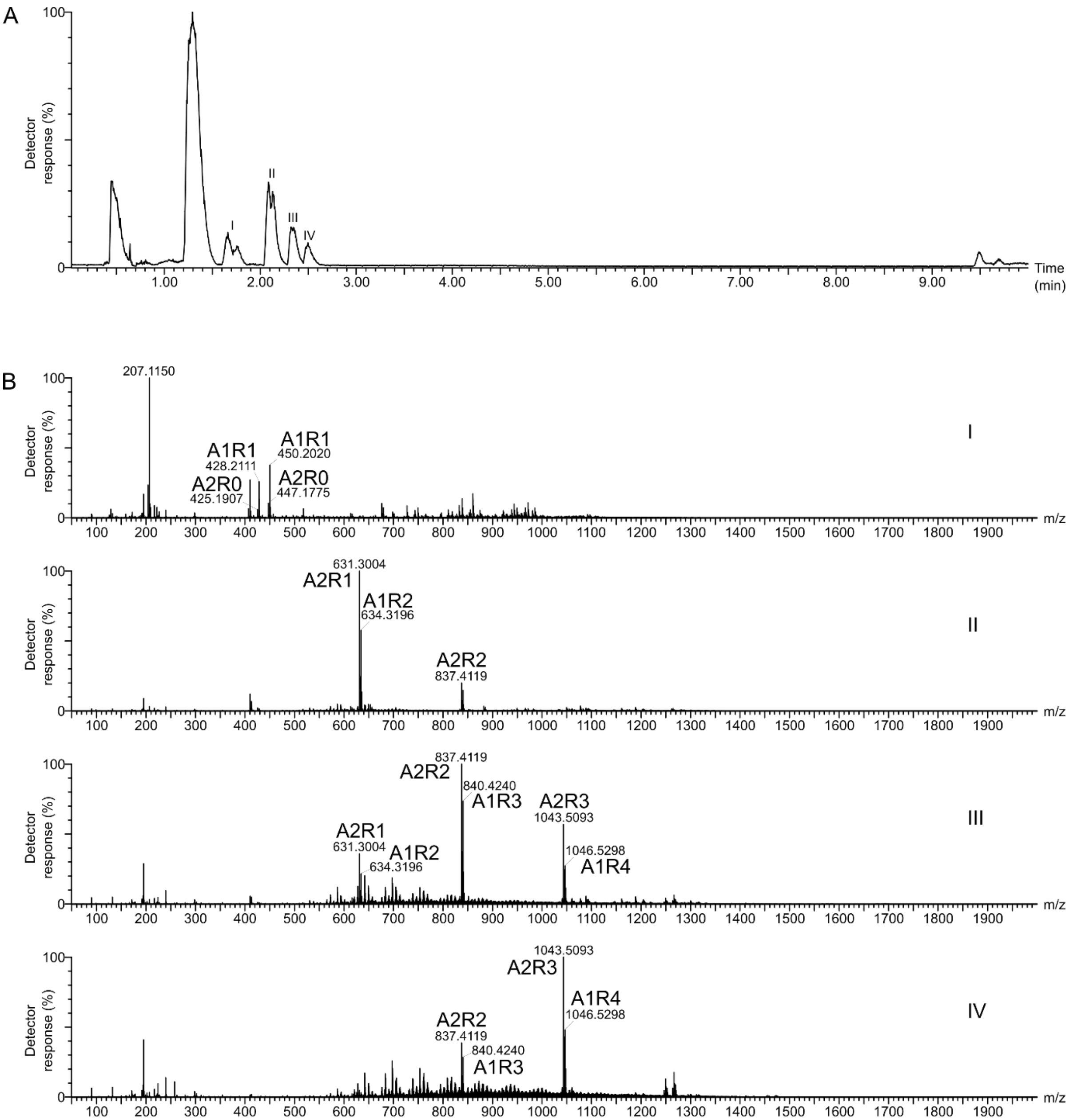

Supplementary Figure 4: Exemplary MS[1] data of the subsite preference analysis of *Si*CHIT[-CBM5] on DA 50% chitosan after 24 h incubation. A) Base peak chromatogram of the HILIC-ESI-MS[1] measurement of the small enzymatic products after *N*-acetylation. **B)** Average mass spectra over each of the peaks labelled **I**-**IV** in **A**, the oligomers are identified based on the m/z value corresponding to the proton or sodium adduct of charge +1. A = acetylated, R = re-acetylated unit (formerly de-acetylated).

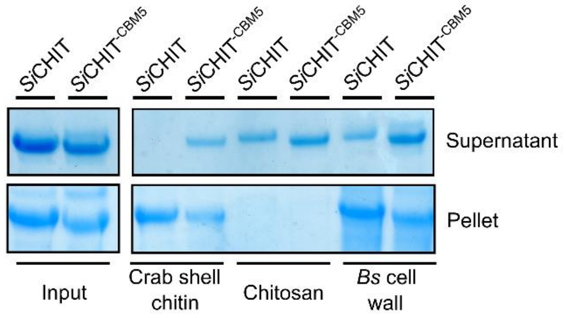

Supplementary Figure 5: CBM5 contributes to substrate binding. 15 mg of crab shell chitin, ≥75 % de-acetylated chitosan or *Bs* cell wall were incubated with 4 µM of recombinant protein for substrate binding. Samples were spun down and protein contents in the supernatant and the substrate pellet were visualized via Coomassie staining following SDS-PAGE. The experiment was repeated with similar results.

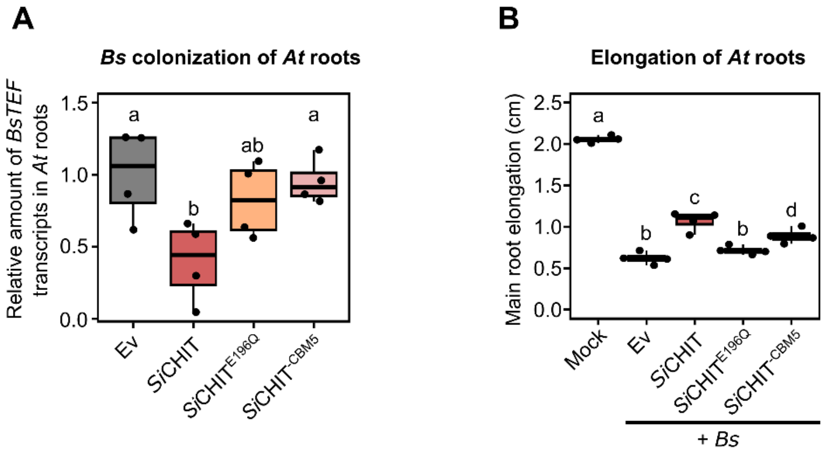

Supplementary Figure 6: The CBM5 is essential to protect *A. thaliana* from the pathogen *Bs*. A) Colonization of *At* roots by *Bs* at three dpi inferred from relative expression of the fungal housekeeping gene *BsTEF* compared to the *At* housekeeping gene *AtUBI* by qPCR using the $2^{-\Delta CT}$ method. Colonization values were normalized to the Ev control. *Bs* spores were either pre-treated with the Ev control or 10 µM of recombinant chitinases for 20 h at 28 °C (mean +/- SD, n = 4). **B)** *At* root elongation at three dpi with *Bs* spores. *Bs* spores were either pre-treated with the Ev control or 10 µM of recombinant chitinases (mean +/- SD, n = 4). Limits of the boxplots represent the 25th–75th percentile, the horizontal line represents the median and the whiskers the minimum/maximum values without outliers. Statistical analysis: Different letters indicate significant differences according to one-way ANOVA followed by Tukey' honest significant difference test (adjusted p-value < 0.05).

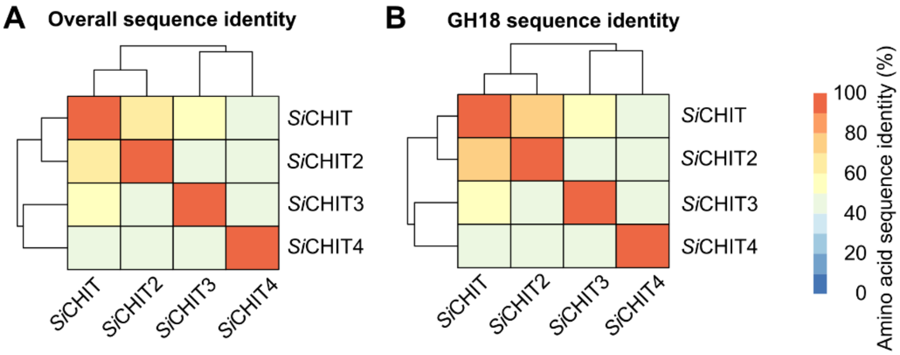

Supplementary Figure 7: Sequence identity of the four *Si* GH18 chitinases. The amino acid sequences without signal peptide were aligned and sequence identity in percent (%) calculated and visualized as heatmap. **A)** Overall sequence identity: the full amino acid sequence without signal peptide was used for the alignment. **B)** GH18 sequence identity: only the amino acid sequence identity of the predicted GH18 domains was used for the alignment.

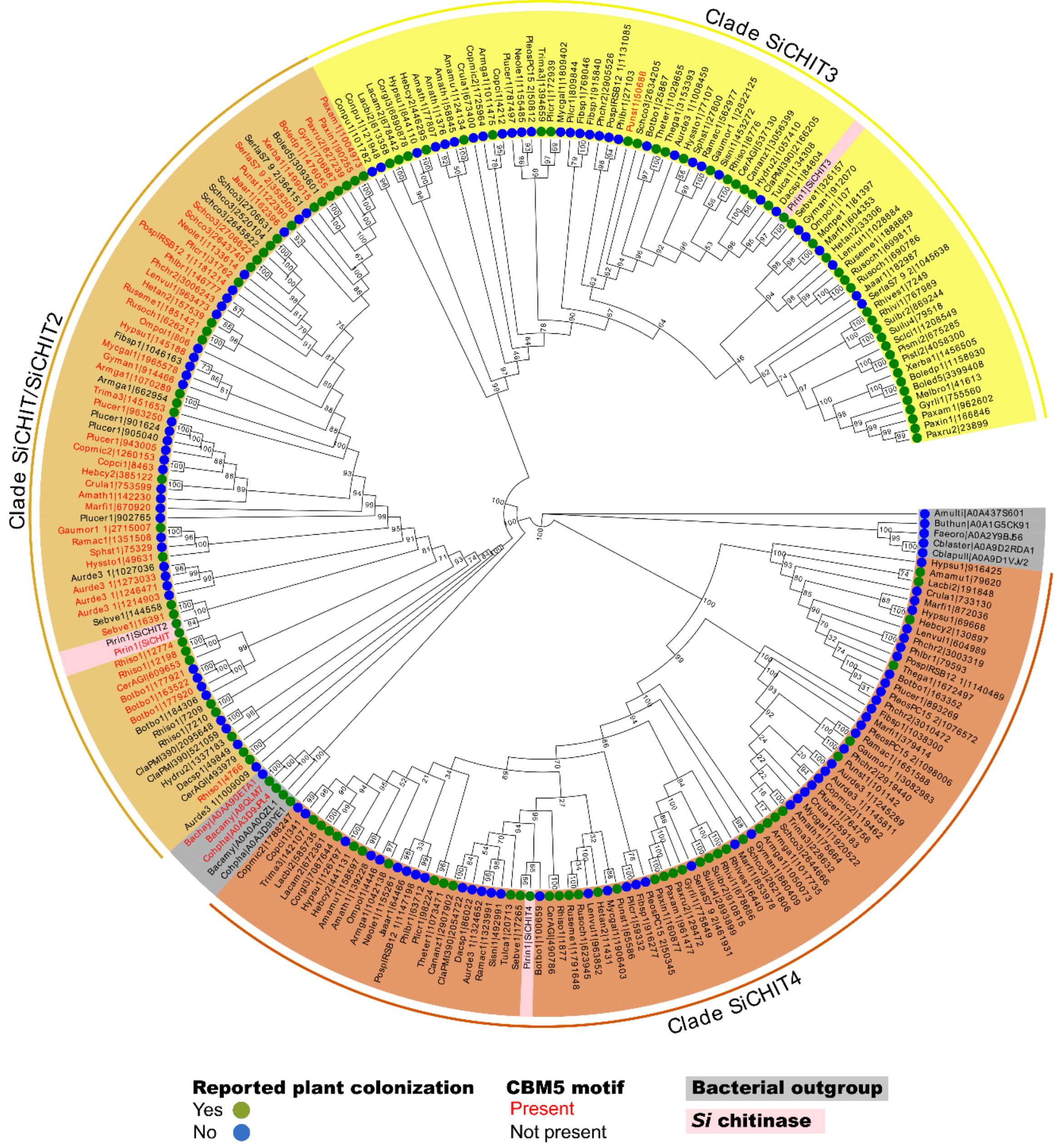

Supplementary Figure 8: Inferred Phylogeny of Fungal GH18-CBM5 Sequences in Basidiomycota. The tree was outputted by IQ-TREE (version 2.0.7). It shows the relation among 230 fungal and 10 bacterial GH18 protein sequences. Only the GH18 domains were used as an input for the software after pre-processing. Each tip corresponds to one sequence. The background colour behind the tip labels indicates whether the sequence is bacterial (grey) or fungal (not grey). *Si* chitinases are specifically highlighted with a light pink background (Pirin1|74346 = *Si*CHIT; Pirin1|74345 = *Si*CHIT2; Pirin1|71855 = *Si*CHIT3; Pirin1|78411 = *Si*CHIT4). Three clades of fungal chitinases are distinguished by different background colours (lightyellow, yellow, or orange). The fill of the tip points reflects whether the corresponding species was identified *in planta* (green) or not (blue). The tip labels contain the species symbol and protein identifier separated by "|". For fungal sequences, the species symbol is the JGI identifier and for bacterial sequences it was chosen manually. The colour of the tip label refers to the presence of CBM5 (red) or absence (black)

in the full-length sequence. The node labels reflect branch support with the bootstrapping result. A branch with a bootstrapping value > 95 is considered supported.

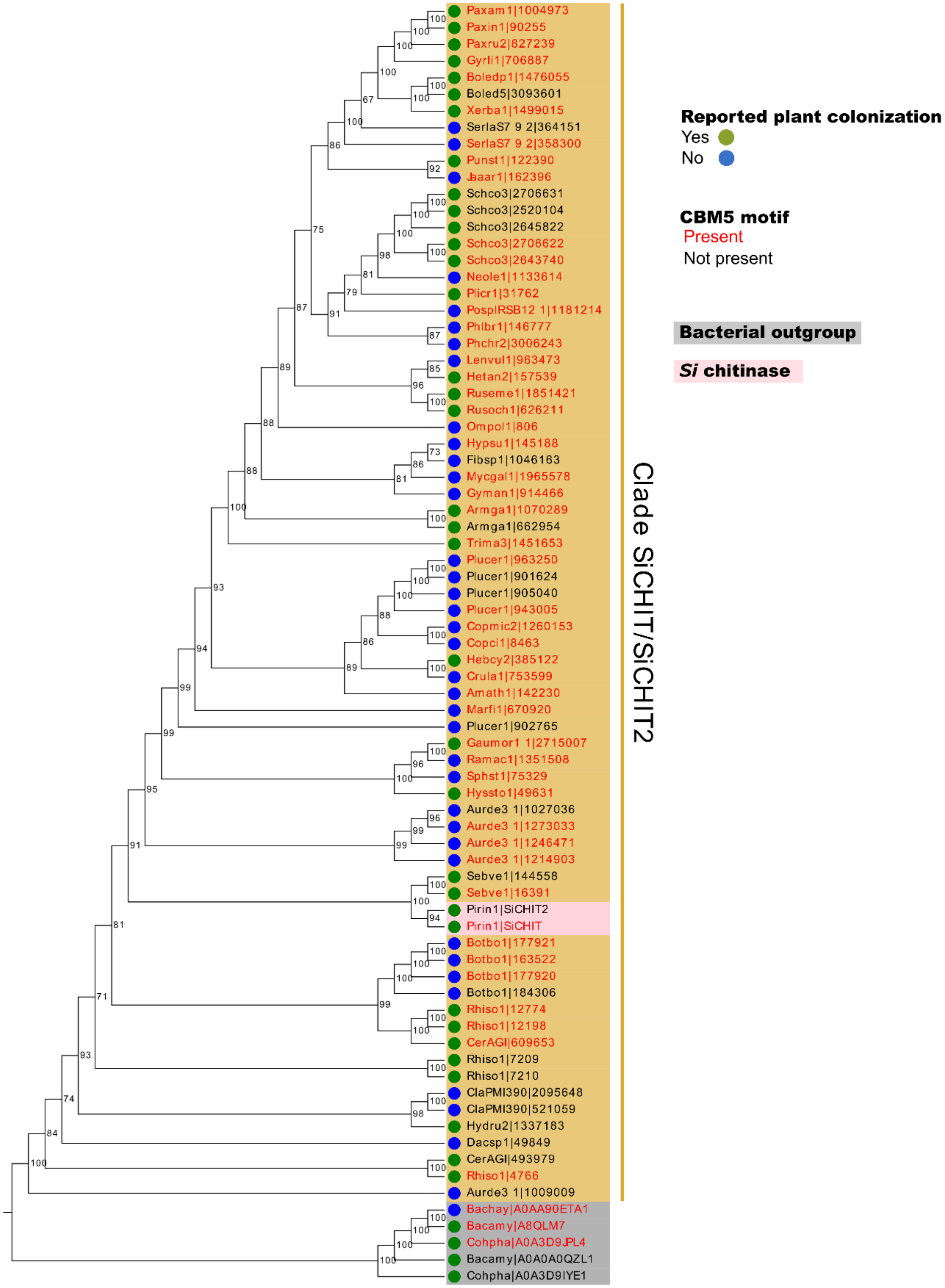

Supplementary Figure 9: Phylogenetic analysis of CBM5-containing chitinases. This tree shows the CBM5-containing branch of the circular tree in Supplementary Figure 8. The tree was outputted by IQ-TREE (version 2.0.7). It shows 72 fungal and 5 bacterial GH18 protein sequences. Only GH18 domains

were used as an input for the software after pre-processing. Each tip corresponds to one sequence. The background colour behind the tip labels indicated whether the sequence is bacterial (grey) or fungal (light yellow). *S. indica* chitinases are specifically highlighter with a light pink background (Pirin1|74346 = *Si*CHIT; Pirin1|74345 = *Si*CHIT2; Pirin1|71855 = *Si*CHIT3; Pirin1|78411 = *Si*CHIT4). The fill of the tip points indicates whether the corresponding species was identified *in planta* (green) or not (blue). The tip labels contain the species symbol and protein identifier separated by "|". For fungal sequences, the species symbol is the JGI identifier and for bacterial sequences it was chosen manually. The colour of the tip label refers to the presence of CBM5 (red) or absence (black) in the full-length sequence. The node labels reflect branch support with the bootstrapping result. A branch with a bootstrapping value > 95 is considered supported.

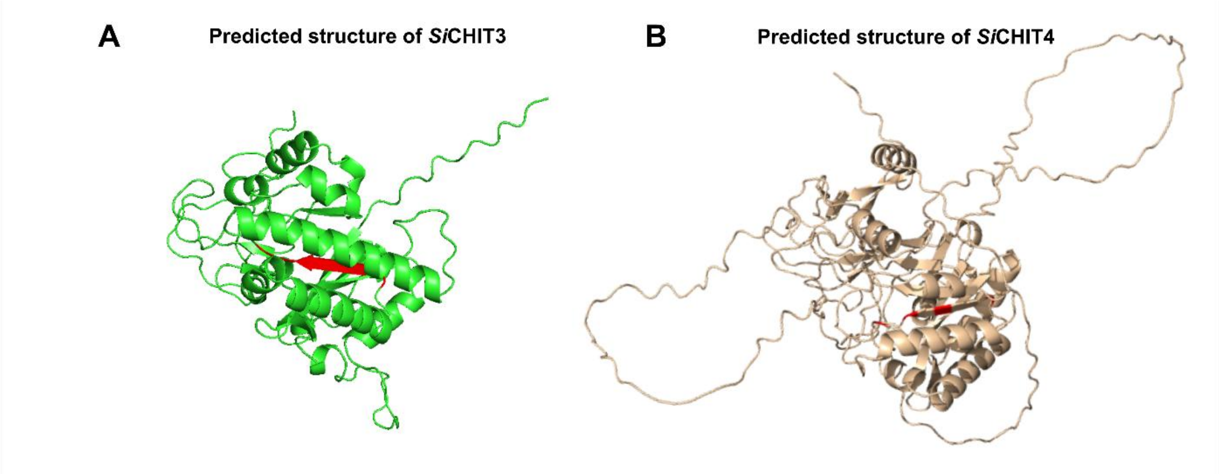

Supplementary Figure 10: Predicted protein structures of *Si*CHIT3 and *Si*CHIT4. A) Structure prediction of *Si*CHIT3. **B)** Structure prediction of *Si*CHIT4. Structures were predicted from amino acid sequences retrieved from NCBI using AlphaFold2 (predicted with ColabFold version 1.5.2). The sequences of predicted signal peptides were identified with SignalP 5.0 and removed prior to structure prediction.

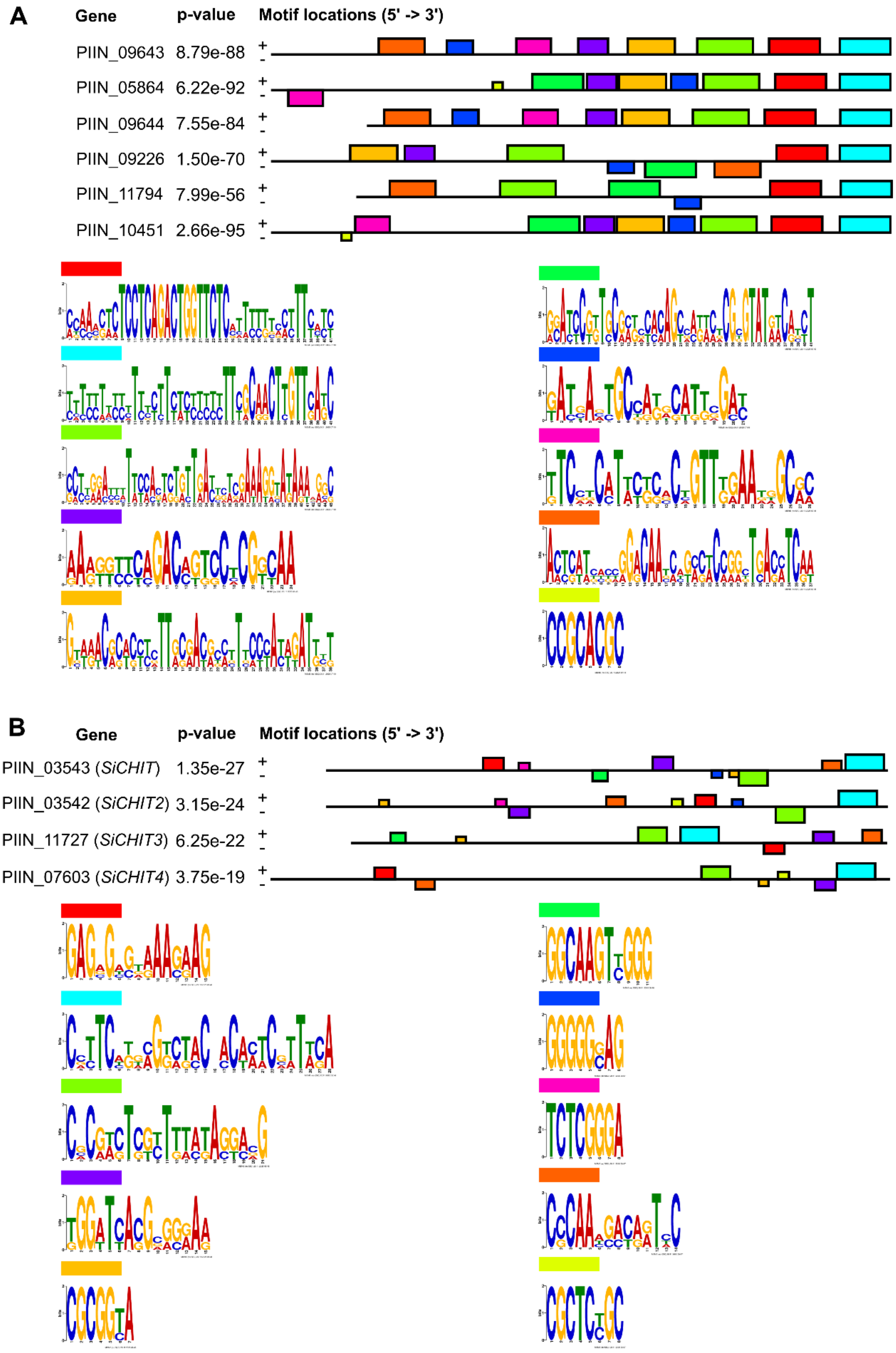
A
Gene
p-value
Motif locations (5' -> 3')
PIIN_09643 8.79e-88
PIIN_05864 6.22e-92
PIIN_09644 7.55e-84
PIIN_09226 1.50e-70
PIIN_11794 7.99e-56
PIIN_10451 2.66e-95
B
Gene
p-value
Motif locations (5' -> 3')
PIIN_03543 (SiCHIT) 1.35e-27
PIIN_03542 (SiCHIT2) 3.15e-24
PIIN_11727 (SiCHIT3) 6.25e-22
PIIN_07603 (SiCHIT4) 3.75e-19

Supplementary Figure 11: *Si* chitinase genes display motif variation in regulatory regions. A) Motif identification in regulatory regions of genes encoding the expanded and conserved DELD family and **B)** the four *Si* chitinases. A maximum of 500 bp 5' upstream of the gene coding regions was chosen for motif identification using the MEME suite online tool. The maximum number of motifs to identify was set to ten. Bar colors above motif logos reflect the positions of the identified motifs.

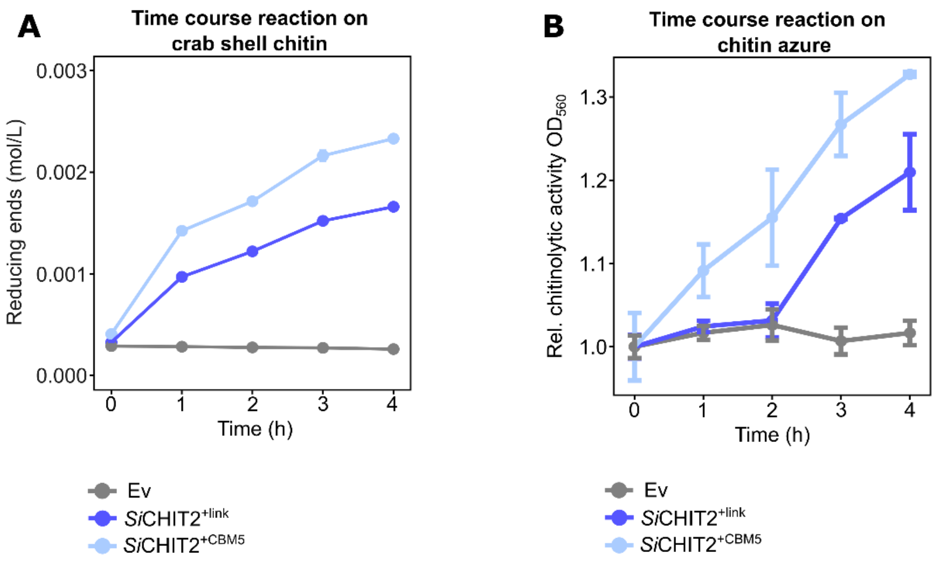

Supplementary Figure 12: Chitinolytic activity of *Si*CHIT2^{+link} and *Si*CHIT2^{+CBM5} at different timepoints. A) Chitinase activity of recombinant *Si*CHIT2^{+link} and *Si*CHIT2^{+CBM5} at 10 µM measured over 4 h at 28 °C. Samples were spun down and supernatants mixed 1:1 with DNSA reagent. Samples were boiled at 100 °C for 10 min and placed on ice. Subsequently, the relative amount of reducing ends was measured at 540 nm (mean +/- SD, n = 3). **B)** Chitinase activity of recombinant *Si*CHIT2^{+link} and *Si*CHIT2^{CBM5} at 10 µM on chitin azure measured over 4 h at 28 °C and normalized to the value of t_0 (mean +/- SEM, n = 3).

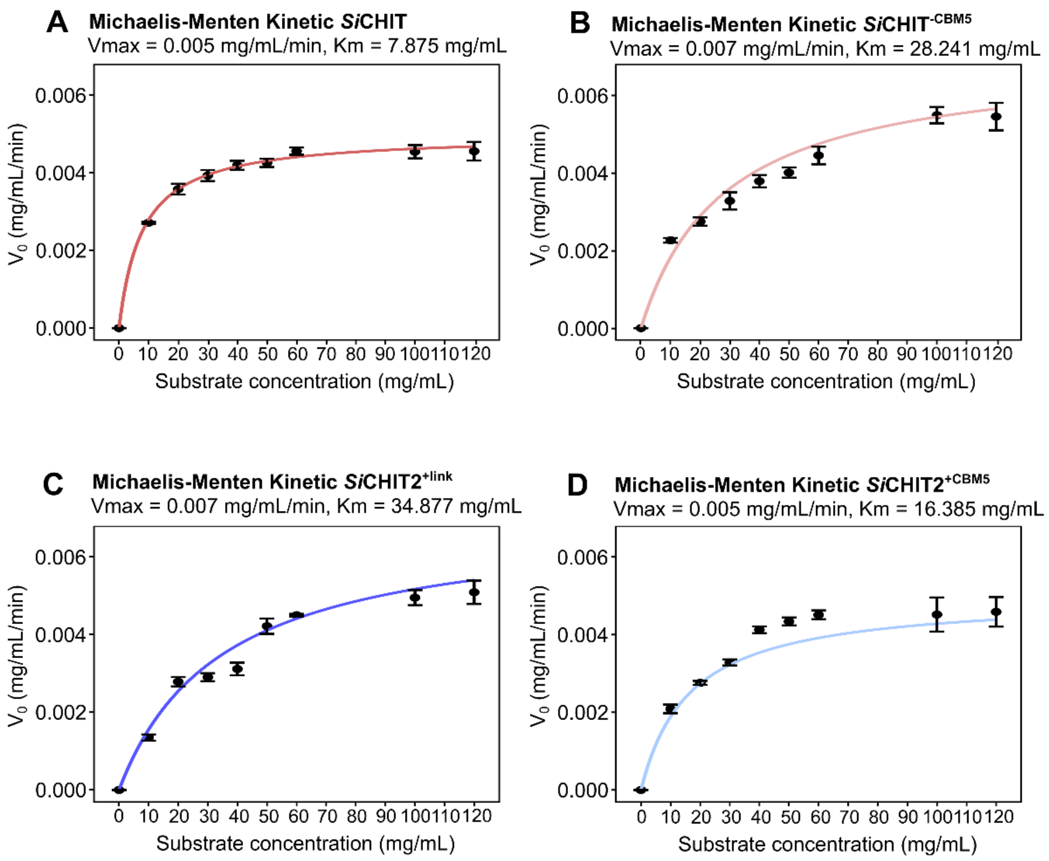

Supplementary Figure 13: Presence of CBM5 affects approximal Michaelis-Menten kinetics of *Si* chitinases on crab shell chitin. A) Kinetic of *Si*CHIT on crab shell chitin. **B)** Kinetic of *Si*CHIT^{-CBM5} on crab shell chitin. **C)** Kinetic of *Si*CHIT2^{+link} on crab shell chitin. **D)** Kinetic of *Si*CHIT2^{+CBM5} on crab shell chitin. 5 µM of recombinant enzyme were incubated with crab shell chitin at 37 °C. Reactions were stopped after 60 min and product formation was assessed using a DNSA assay. Curve-fitting and Michaelis-Menten kinetics were calculated using the renz and drc package in R (mean +/- SD, n = 3).

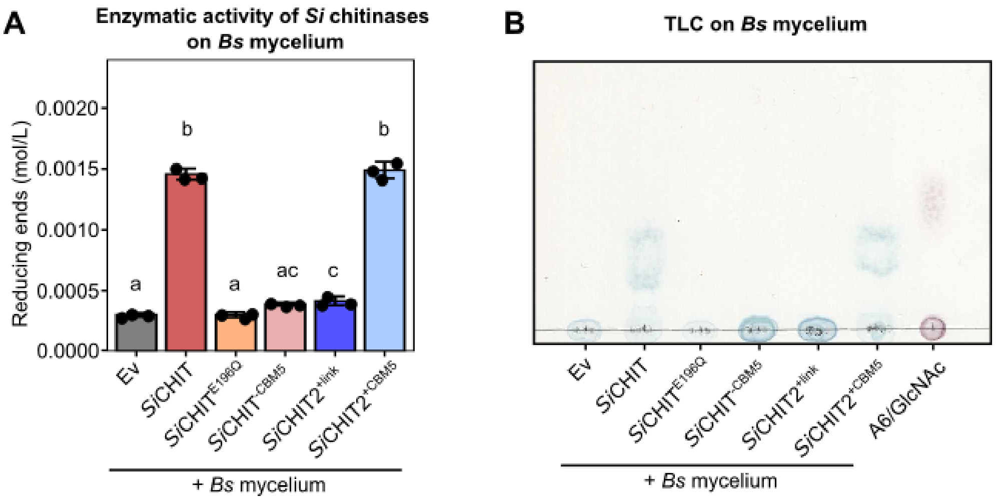

Supplementary Figure 14: Activity of *Si* chitinases on *Bs* mycelium is enhanced by CBM5. A) Release of reducing ends from lyophilized *Bs* mycelium treated with recombinant chitinases at 10 μM for 16 h at 28 °C. Samples were spun down and supernatants mixed 1:1 with DNSA reagent. Samples were boiled at 100 °C for 10 min and placed on ice. Subsequently, the relative amount of reducing ends was measured at 540 nm (mean +/- SD, n = 3). Statistical analysis: Different letters indicate significant differences according to one-way ANOVA followed by Tukey' honest significant difference test (adjusted p-value < 0.05). **B)** Thin layer chromatography (TLC) of hydrolysates of *Bs* mycelium treated with recombinant chitinases at 10 μM for 16 h at 28 °C and loaded on a silica plate for TLC. The experiment was repeated with similar results.

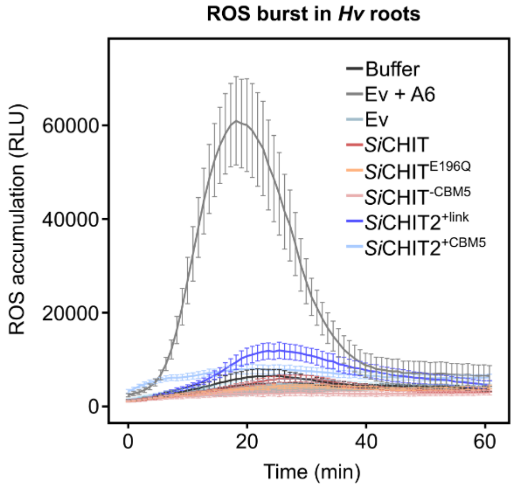

Supplementary Figure 15: ROS burst in Hv roots. ROS burst of 4 d *Hv* roots after treatment with 125 nM chitohexaose. 10 µM chitohexaose (A6) were incubated with the Ev control 20 h prior to treatment of *Hv* roots. Roots were treated with the indicated elicitors at 125 nM. Ev + A6 and Buffer values from Fig. 5A were used for proper comparison with the treatments (mean +/- SEM, n = 5).

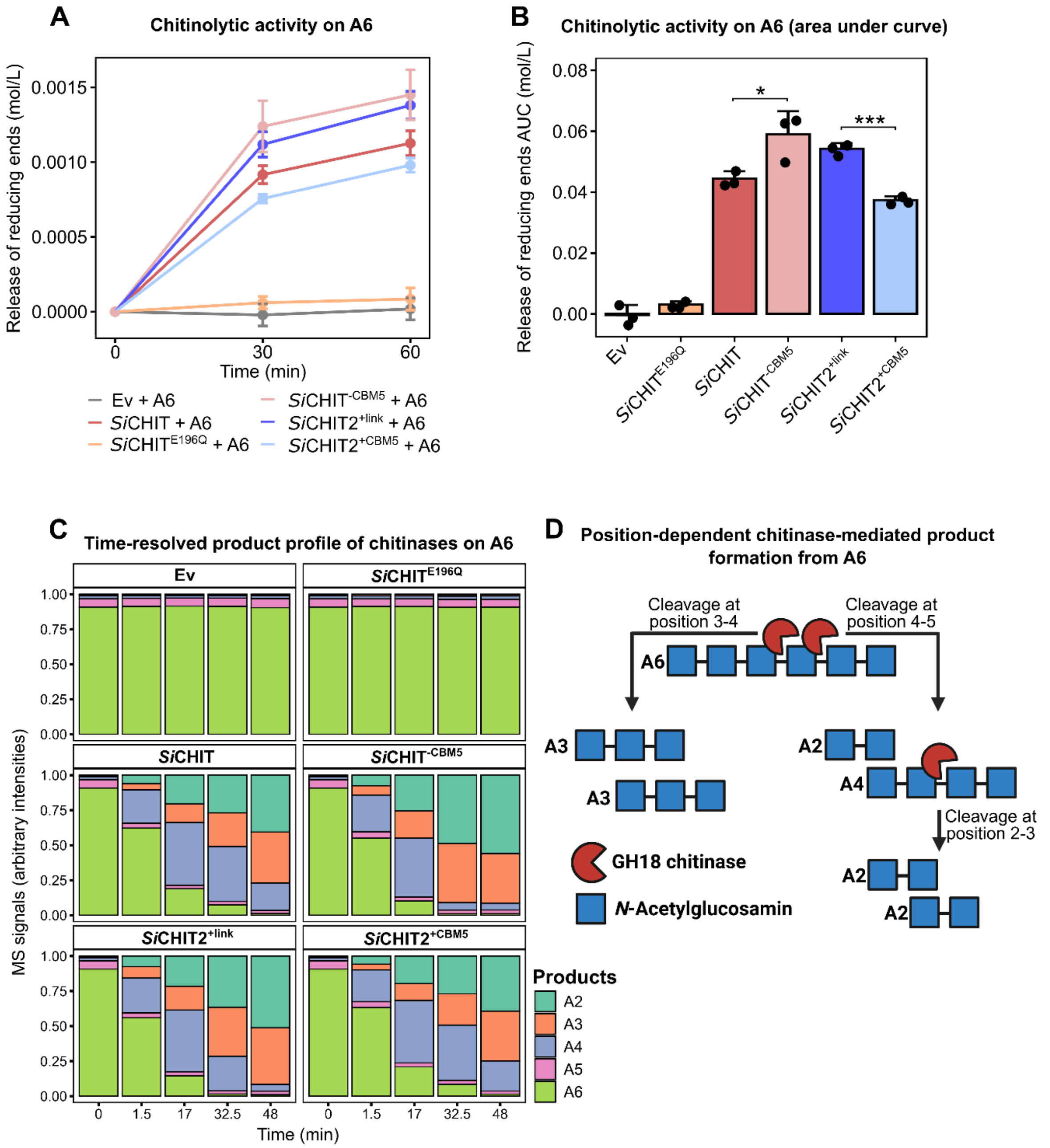

Supplementary Figure 16: Chitinolytic activity on immunogenic chitohexaose. A) Chitinolytic activity of all recombinant chitinases 30 and 60 min on chitohexaose (A6). 600 nM of A6 was incubated with an equimolar concentration of chitinases and measured at 0, 30, and 60 min. Samples were mixed 1:1 with DNSA reagent, boiled at 100 °C for 10 min and placed on ice. Absorbance measurements were taken at 540 nm. Values for t = 0 were subtracted from values for t = 30 min and t = 60 min (mean +/- SD, n = 3). **B)** Calculation of the area under the curve (AUC) from **A** (mean +/- SD, n = 3). Statistical differences were inferred by two-sided student's t-test (P-value: * < 0.05; ** < 0.01). **C)** Time-resolved mass spectrometric of A6 incubated with different recombinant chitinase variants, monitored by live SEC-RI-MS at 37 °C. Stacked bars represent relative MS signal intensities of oligomeric products (A2-A6) over time. **D)** Cleavage pattern of chitohexaose (A6) by GH18 chitinases inferred from SEC-RI-MS. Depending on the cleavage position on A6, GH18 chitinases produce two molecules A3 or one molecule A2 and one molecule A4. A4 can be further cleaved into two molecules A2. The scheme was produced using BioRender *(https://BioRender.com/l75bx0s.)*

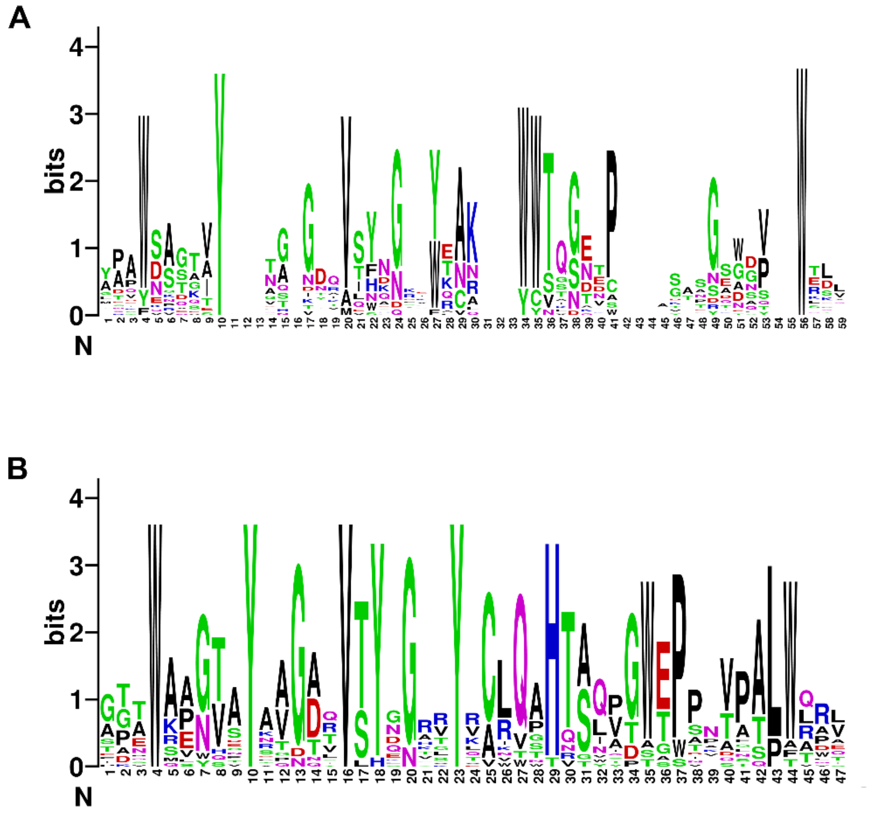

Supplementary Figure 17: CBM5 vs CBM12. Sequence logos for **A)** CBM5 and **B)** CBM12 motifs are shown. The logos were generated using WebLogo from representative CBM5 or 12 sequences from the CAZy database.

Supplementary Table 1: Domain composition of the four GH18 chitinases from *Si*.

Protein ID	No. amino acids	Signal peptide	GH18 domain	CBM5	Remarks
PIIN_03543	533	1-19	61-405	486-530	C-terminal CBM5
PIIN_03542	495	1-16	65-409	None	No CBM5 but C-terminal linker
PIIN_11727	314	None	20-311	None	No signal peptide
PIIN_07603	523	1-28	155-437	None	Three low complex complexity loops within the GH18<br>a: 154-167<br>b: 286-297<br>c: 406-417

Supplementary Table 2: Primers used in the study.

Primer name	Sequence 5' → 3'	Use
PIIN_03543_qPCR_for	cctgggtcttgggagaatg	qPCR
PIIN_03543_qPCR_rev	ggcgtcgtagcagtatgaa	qPCR
PIIN_03542_qPCR_F	acggcatctgggactacaaa	qPCR
PIIN_03542_qPCR_R	gcagaaagttcccagtgcat	qPCR
PIIN_11727_qPCR_for	cctttcgttctggaacctgat	qPCR
PIIN_11727_qPCR_rev	cgactgccatcgtggtattta	qPCR
PIIN_07603_qPCR_FW1	gatgttcgggagtttgcgag	qPCR
PIIN_07603_qPCR_RV1	aatcttctggtgcggcattc	qPCR
HvPR10_FW (Sakar et al 2019)	ggagggcgacaaggtaagtg	qPCR
HvPR10_RV (Sakar et al 2019)	cgtccagcctctcgtactct	qPCR
Bs_Tef_for (Sakar et al. 2019)	cgccgtaccggaaagtctg	qPCR
Bs_Tef_rev (Sakar et al. 2019)	ggcgaaacgaccaagagga	qPCR
TEF_Piri_QPCR_F	gcaagttctccgagctcatc	qPCR
TEF_Piri_QPCR_R	ccaagtggtgggtactcgtt	qPCR
HvUbi60_fwd (Sakar et al. 2019)	accctcgccgactacaacat	qPCR
HvUbi60_rev (Sakar et al. 2019)	cagtagtggcggtcgaagtg	qPCR
SiCHIT_pQE_FW	atcaccatcaccatcacggatccgcatgcgagctcggtaccacgcccgccatgatgc	cloning
SiCHIT_pQE_RV	ctcagctaattaagcttggctgcaggtcgacccggggtacctcagcacgacttgagttgattccac	cloning
SiCHITtrun_pQE_RV	ctcagctaattaagcttggctgcaggtcgacccggggtacctcacataccagcacatgttccacc	cloning
PIIN_03542_cloning_F	tccgcatgcgagctcggtaccgtcgttggacgtcccaagaa	cloning
PIIN_03542_cloning_R	agccccttttgtctgcagaaagttcccag	cloning
PIIN_03542_Linker_cloning_F	ttctgcagacaaaaggggctccgagtcg	cloning
PIIN_03542_Linker_cloning_R	tgcaggtcgacccggggtacctcacataccagcacatgttccacc	cloning
PIIN_03542_Linker_CBM5_F	ttctgcagacaaaaggggctccgagtc	cloning
PIIN_03542_Linker_CBM5_R	tgcaggtcgacccggggtacctcagcacgacttgagttgattccacg	cloning
