# Supplementary material for: Domain gain or loss in a fungal chitinase enables specialization towards antagonism or immune suppression
Source: Nat Commun. 2026 Mar 30;17:3115. doi: 10.1038/s41467-026-71064-0 (PMC13039781; doi:10.1038/s41467-026-71064-0)
Supplement: Supplementary file 2 — Description of Additional Supplementary Files [file 41467_2026_71064_MOESM2_ESM.pdf]

Description of Additional Supplementary Files

File Name: Supplementary Data 1

Description: The phylogeny data used in this study.

Sheet Name	Content
01. Supplementary Sheet Guide	This has the orientation to navigate the supplementary file
02. List of Species Included JGI	This has the list of fungal species that were in the tree construction. All GH18 sequences from these species were used
03. Pirin1 Sebve1 GH18 Domains	This has the extracted GH18 domains (according to Mycocosm annotation) from all S.indica (Pirin1) and S.vermifera (Sebve1) GH18 chitinases
04. Full Fungal Seq HMM Results	Here are the results of the hmmsearch of Pirin1 and Sebve1 GH18 hmm profile. The full sequences are shown as well as the best domain score from the hmm search as well as the start and ending position of the GH18 domain.
05. Full Fungal Seq HMM Results	Here are the GH18 domains of the fungal sequences that had a best domain score more than 200. The GH18 domains were extracted using the starting and ending positions from the hmmsearch
06. Bacterial GH18 HMM score	Here are the GH18 domains of the selected bacterial outgroup sequences that were extracted the same way the fungal GH18 domains were extracted. In addition you find the best domain score from the hmmsearch of the Pirin1 and Sebve1 hmm profile
07. Annotation File for Input	This is an annotation file that shows each sequence on the tree, the corresponding , and other attributes like taxonomy, presence of CBM5 motif, lifestyle of fungus, and more
08. Complete Tree Newick	This shows the complete tree in Newick format

09. CBM5 Subtree Newick	This shows the CBM5-containing subtree in Newick format
10. Classification CBM5 or CBM12	This shows the classification of the sequences of that were annotated to contain CBM5/12 motif and were featured in the tree. The best domain score of the hmmsearch of CBM5 or CBM12 profiles are shown. All sequences featured have CBM5 HMM score higher than the CBM12 one
11. CBM5 domains	These were the CBM5 domains used in contructing the CBM5 HMM profile and logo
12. CBM12 domains	These were the CBM12 domains used in contructing the CBM12 HMM profile and logo
13. CBM12 Score Validation	These were the CBM12 sequences that had the CBM12 and CBM5 HMM score tested on them. These sequence had a higher CBM12 score than the corresponding CBM5 score
14. CBM5 Score Validation	These were the CBM5 sequences that had the CBM12 and CBM5 HMM score tested on them. These sequence had a higher CBM5 score than the corresponding CBM12 score
