# Supplementary material for: Domain gain or loss in a fungal chitinase enables specialization towards antagonism or immune suppression
Source: Nat Commun. 2026 Mar 30;17:3115. doi: 10.1038/s41467-026-71064-0 (PMC13039781; doi:10.1038/s41467-026-71064-0)
Supplement: Supplementary file 4 — Reporting Summary [file 41467_2026_71064_MOESM4_ESM.pdf]

nature portfolio

Corresponding author(s): Alga Zuccaro

Last updated by author(s): Mar 3, 2026

Reporting Summary

Nature Portfolio wishes to improve the reproducibility of the work that we publish. This form provides structure for consistency and transparency in reporting. For further information on Nature Portfolio policies, see our Editorial Policies and the Editorial Policy Checklist.

Statistics

For all statistical analyses, confirm that the following items are present in the figure legend, table legend, main text, or Methods section.

n/a	Confirmed	
☐	☒	The exact sample size (*n*) for each experimental group/condition, given as a discrete number and unit of measurement
☒	☐	A statement on whether measurements were taken from distinct samples or whether the same sample was measured repeatedly
☐	☒	The statistical test(s) used AND whether they are one- or two-sided<br>*Only common tests should be described solely by name; describe more complex techniques in the Methods section.*
☒	☐	A description of all covariates tested
☒	☐	A description of any assumptions or corrections, such as tests of normality and adjustment for multiple comparisons
☐	☒	A full description of the statistical parameters including central tendency (e.g. means) or other basic estimates (e.g. regression coefficient) AND variation (e.g. standard deviation) or associated estimates of uncertainty (e.g. confidence intervals)
☐	☒	For null hypothesis testing, the test statistic (e.g. *F*, *t*, *r*) with confidence intervals, effect sizes, degrees of freedom and *P* value noted<br>*Give P values as exact values whenever suitable.*
☒	☐	For Bayesian analysis, information on the choice of priors and Markov chain Monte Carlo settings
☒	☐	For hierarchical and complex designs, identification of the appropriate level for tests and full reporting of outcomes
☒	☐	Estimates of effect sizes (e.g. Cohen's *d*, Pearson's *r*), indicating how they were calculated

Our web collection on statistics for biologists contains articles on many of the points above.

Software and code

Policy information about availability of computer code

Data collection	Amino acid sequence data were retrieved from the NCBI server or the Mycocosm server. All other data were obtained experimentally. Gene expression data via qPCR were obtained using the Biorad CFX96 Connect system. Absorbance and chemiluminescence data were obtained with a TECAN plate reader and the Sparkcontrol V2.1 software.
Data analysis	Data was analysed using the R software version 4.1.1 (2021-08-10) -- "Kick Things" and Microsoft Excel.<br>ColabFold v1.5.5: AlphaFold2 using MMseqs2 was used to predict protein structures.<br>Phylogenetic analyses were conducted using MEGA version X, MAAFT (v7), Clipkit (v2.6.1), IQ-TREE (v2.0.7), Evolview (v2)

For manuscripts utilizing custom algorithms or software that are central to the research but not yet described in published literature, software must be made available to editors and reviewers. We strongly encourage code deposition in a community repository (e.g. GitHub). See the Nature Portfolio guidelines for submitting code & software for further information.

Data

Policy information about availability of data

All manuscripts must include a data availability statement. This statement should provide the following information, where applicable:

- Accession codes, unique identifiers, or web links for publicly available datasets
- A description of any restrictions on data availability
- For clinical datasets or third party data, please ensure that the statement adheres to our policy

All experimental data used in this study are provided in the source data file. The phylogeny data used in this study are available in the Supplementary Data 1 file.

Research involving human participants, their data, or biological material

Policy information about studies with human participants or human data. See also policy information about sex, gender (identity/presentation), and sexual orientation and race, ethnicity and racism.

Reporting on sex and gender	N/A
Reporting on race, ethnicity, or other socially relevant groupings	N/A
Population characteristics	N/A
Recruitment	N/A
Ethics oversight	N/A

Note that full information on the approval of the study protocol must also be provided in the manuscript.

Field-specific reporting

Please select the one below that is the best fit for your research. If you are not sure, read the appropriate sections before making your selection.

☒ Life sciences ☐ Behavioural & social sciences ☐ Ecological, evolutionary & environmental sciences

For a reference copy of the document with all sections, see nature.com/documents/nr-reporting-summary-flat.pdf

Life sciences study design

All studies must disclose on these points even when the disclosure is negative.

Sample size	No estimation of sample size has been conducted. Sample sizes were chosen on previous experience in the lab.
Data exclusions	No data was excluded.
Replication	At least three independent replications for biochemical assays (chitin azure, DNSA, protein binding, subsite preferende) and at least four independent replications for fungal infection assays (chitinase-mediated protection, fungal spore germination, chitinase-mediated immune modulation, chitinase expresssion during confrontation. Replications were carried out with independently grown fungal strains and independent batches of recombinant protein. Replication attempts were succesful.
Randomization	Incubated plants were grown randomly allocated in the same growth chamber. Fungal samples treated with recombinant enzymes were placed randomly in the same incubator.
Blinding	Blinding was not relevant to our study.

Reporting for specific materials, systems and methods

We require information from authors about some types of materials, experimental systems and methods used in many studies. Here, indicate whether each material, system or method listed is relevant to your study. If you are not sure if a list item applies to your research, read the appropriate section before selecting a response.

Materials & experimental systems

n/a	Involved in the study
☒	☐ Antibodies
☒	☐ Eukaryotic cell lines
☒	☐ Palaeontology and archaeology
☒	☐ Animals and other organisms
☒	☐ Clinical data
☒	☐ Dual use research of concern
☐	☒ Plants

Methods

n/a	Involved in the study
☒	☐ ChIP-seq
☒	☐ Flow cytometry
☒	☐ MRI-based neuroimaging

Dual use research of concern

Policy information about dual use research of concern

Hazards

Could the accidental, deliberate or reckless misuse of agents or technologies generated in the work, or the application of information presented in the manuscript, pose a threat to:

No	Yes
☒	☐ Public health
☒	☐ National security
☒	☐ Crops and/or livestock
☒	☐ Ecosystems
☒	☐ Any other significant area

Experiments of concern

Does the work involve any of these experiments of concern:

No	Yes
☒	☐ Demonstrate how to render a vaccine ineffective
☒	☐ Confer resistance to therapeutically useful antibiotics or antiviral agents
☒	☐ Enhance the virulence of a pathogen or render a nonpathogen virulent
☒	☐ Increase transmissibility of a pathogen
☒	☐ Alter the host range of a pathogen
☒	☐ Enable evasion of diagnostic/detection modalities
☒	☐ Enable the weaponization of a biological agent or toxin
☒	☐ Any other potentially harmful combination of experiments and agents

Plants

Seed stocks	Hordeum vulgare Golden-Promise seeds were received fom the IPK Gatersleben and propagated and stored in our laboratory. Arabidopsis thaliana Col-0 is stored at our institute at the University of Cologne.
Novel plant genotypes	NIA
Authentication	N/A
